# Analysis of the Complete Mycoplasma hominis LBD-4 Genome Sequence Reveals Strain-Variable Prophage Insertion and Distinctive Repeat-Containing Surface Protein Arrangements

**DOI:** 10.1128/genomeA.01582-14

**Published:** 2015-02-26

**Authors:** Michael J. Calcutt, Mark F. Foecking

**Affiliations:** Department of Veterinary Pathobiology, University of Missouri, Columbia, Missouri, USA

## Abstract

The complete genome sequence of Mycoplasma hominis LBD-4 has been determined and the gene content ascribed. The 715,165-bp chromosome contains 620 genes, including 14 carried by a strain-variable prophage genome related to Mycoplasma fermentans MFV-1 and Mycoplasma arthritidis MAV-1. Comparative analysis with the genome of M. hominis PG21^T^ reveals distinctive arrangements of repeat-containing surface proteins.

## GENOME ANNOUNCEMENT

Mycoplasma hominis is primarily considered a urogenital tract inhabitant (1), but multiple reports of extragenital infections highlight the multifarious tropisms and clinical manifestations of this organism (2, 3). The complete genome sequence of M. hominis PG21^T^ (isolated from the female lower genital tract) has been determined (4), and a draft genome sequence of 22 contigs was recently released for blood sample isolate M. hominis LBD-4 as part of the one thousand microbial genomes project (5). This isolate was recovered from a septicemic patient in 1965 (6). To gain further insight into overall conservation of genomic organization between M. hominis isolates and to determine the variations in the repeated peptide sequences that are present in multiple large M. hominis surface proteins, the complete genome of M. hominis LBD-4 was determined.

Genomic DNA was prepared from a late-logarithmic-phase culture of M. hominis LBD-4 (obtained from the American Type Culture Collection as ATCC 27545), further purified using a PowerClean Pro DNA cleanup kit (Mo Bio Laboratories, CA), and sequenced using Pacific Biosciences chemistry at the National Center for Genome Resources, Santa Fe, NM. A fully assembled genome of 715,165 bp, with a read depth of 616×, was obtained using HGAP version 2 (7) on the reads generated from one single-molecule real-time (SMRT) cell. The complete genome has a 26.94% G+C content and is ~31 kb larger than the previously determined Illumina data set (22 contigs), largely due to the presence of repetitive sequences that were successfully resolved using Pacific Biosciences sequencing. Open reading frames (ORFs) and RNA features were identified using the Prokka tool (8), with the subsequent manual curation of the 620 genes, including the verification of mutations and deletions associated with 16 pseudogenes. The coding sequences comprised those for 563 ORFs, 6 rRNAs (two dispersed 16S-23S rRNA operons and two unlinked 5S rRNA genes), 33 tRNAs, and the *rnpB* and *ssrA* small RNAs.

A preliminary comparative analysis with the 665,445-bp genome of M. hominis PG21^T^ disclosed colinear chromosomes punctuated by ~78 kb of variation distributed among 20 loci (>250 bp); the largest region of difference was an approximately 15.9-kb prophage genome (designated MHoV-1) related to Mycoplasma arthritidis MAV-1 (9) and Mycoplasma fermentans MFV-1 (10). In M. hominis LBD-4, MHoV-1 comprises 14 similarly oriented ORFs and is inserted immediately downstream of an ABC transporter gene.

The use of long-read sequencing enabled the assembly of regions encoding large, peptide-repeat-harboring surface proteins. Size and sequence variations were evident for the multiple LMP repeat-containing surface proteins (4, 11) present in each strain. Furthermore, an expanded array of four genes encoding repeat-containing lipoproteins is present at a locus in M. hominis LBD-4; the corresponding site in strain PG21^T^ encodes a single, albeit larger, repeat-containing lipoprotein gene (MHO_2260).

The availability of a second complete M. hominis genome further informs our understanding of strain divergence in this taxon, with variable mobile genetic element repertoires and genes that are predicted to confer distinctive surface peptide displays encoded within a largely conserved genomic framework.

### Nucleotide sequence accession number.

This complete genome sequence has been deposited at DDBJ/EMBL/GenBank under the accession no. CP009652.
